# Kardiovaskuläre Erkrankungen und Herzinsuffizienz bei Diabetes: Diagnostik und Management (Update 2026)

**DOI:** 10.1007/s00508-025-02667-8

**Published:** 2026-04-30

**Authors:** Matthias W. Heinzl, Michael Resl, Julian Maier, Clemens Steinwender, Jürgen Falkensammer, Paul Fellinger, Thomas Steinmaurer, Christoph H. Saely, Friedrich Hoppichler, Harald Stingl, Thomas C. Wascher, Yvonne Winhofer, Harald Sourij, Bernhard Paulweber, Hermann Toplak, Heinz Drexel, Bernhard Föger, Thomas Stulnig, Martin Clodi

**Affiliations:** 1 Abteilung für Innere Medizin, Konventhospital Barmherzige Brüder Linz, Linz, Österreich; 2 Klinik für Kardiologie und Internistische Intensivmedizin, Kepler Universitätsklinikum Linz, Linz, Österreich; 3 Abteilung für Gefäßchirurgie, Konventhospital Barmherzige Brüder Linz, Linz, Österreich; 4 VIVIT Institut, Akademisches Lehrkrankenaus Feldkirch, Feldkirch, Österreich; 5 Private Universität im Fürstentum Liechtenstein, Triesen, Liechtenstein; 6 Abteilung für Innere Medizin I, Akademisches Lehrkrankenhaus Feldkirch, Feldkirch, Österreich; 7 Abteilung für Innere Medizin, Krankenhaus der Barmherzigen Brüder Salzburg, Salzburg, Österreich; 8 Abteilung für Innere Medizin, Landesklinikum Baden, Baden, Österreich; 9 1. Medizinische Abteilung, Hanusch-Krankenhaus Wien, Wien, Österreich; 10 Universitätsklinik für Innere Medizin III, Endokrinologie und Stoffwechsel, Medizinische Universität Wien, Wien, Österreich; 11 Universitätsklinik für Innere Medizin, Klinische Abteilung für Endokrinologie und Stoffwechsel, Kardiometabolische Trials Unit, Medizinische Universität Graz, Graz, Österreich; 12 Universitätsklinik für Innere Medizin I, mit Gastroenterologie, Hepatologie, Nephrologie, Stoffwechsel und Diabetologie, Paracelsus Medizinische Privatuniversität, Salzburg, Österreich; 13 Universitätsklinik für Innere Medizin, Medizinische Universität Graz, Graz, Österreich; 14 3. Medizinische Abteilung und Karl Landsteiner Institut für Stoffwechselerkrankungen und Nephrologie, Klinik Hietzing, Wien, Österreich; 15 Klinisches Forschungsinstitut für Kardiometabolische Forschung, Johannes Kepler Universität Linz, Linz, Österreich; 16 Abteilung für Innere Medizin, Fachklinik Schwaben, Bad Mergentheim, Deutschland; 17 Emc2-Stoffwechsel- und Studienzentrum, Mattersburg, Österreich

**Keywords:** Diabetes mellitus, Herzinsuffizienz, Koronare Herzkrankheit, Herzinsuffizienz-Screening, Kardiovaskuläres Management, Diabetes mellitus, Heart failure, Coronary artery disease, Heart failure screening, Cardiovascular management

## Abstract

Die wechselseitigen Beziehungen von Diabetes mellitus, kardiovaskulärer Erkrankung und Herzinsuffizienz erfordern einen ganzheitlichen Behandlungsansatz. Bei der Erstmanifestation einer kardiovaskulären Erkrankung sollte stets ein aktives Screening auf Diabetes mellitus erfolgen. Umgekehrt muss bei bereits diagnostiziertem Diabetes mellitus die kardiovaskuläre Risikostratifizierung unter Berücksichtigung aller relevanten Risikofaktoren, Biomarker sowie des klinischen Gesamtbildes erfolgen und um ein optimales individuell abgestimmtes Management zu gewährleisten. Ein interdisziplinäres Vorgehen, das diabetologische, kardiologische und weitere Fachkompetenzen integriert, ist unerlässlich, um kardiovaskuläre Komplikationen bei Menschen mit Diabetes frühzeitig zu erkennen und effektiv zu behandeln.

## Abstract (en)

The interplay between diabetes mellitus, cardiovascular disease and heart failure requires a holistic treatment approach. At the first manifestation of any cardiovascular disease, active screening for diabetes mellitus should always be performed. Conversely, in patients with established diabetes mellitus cardiovascular risk stratification must comprise relevant risk factors, biomarkers and the clinical situation to ensure an optimal individualized management. The integration of diabetological, cardiological and additional specialist expertise is essential to detect cardiovascular complications early in people with diabetes and to treat them effectively.

## Diabetes und kardiovaskuläre Erkrankung

Diabetes mellitus ist ein entscheidender Risikofaktor für kardiovaskuläre Erkrankungen, und kardiovaskuläre Erkrankungen sind wiederum eine führende Ursache für Morbidität und Mortalität bei Menschen mit Diabetes mellitus [1–5]. Die Prävalenz kardiovaskulärer Erkrankungen bei Diabetes mellitus steigt stark mit zunehmendem Lebensalter [6], gleichzeitig geht eine frühere Diagnose von Diabetes mellitus Typ 2 (T2D) mit einem höheren Verlust an erwartbaren Lebensjahren einher [3, 7]. Neben Diabetes mellitus spielen auch häufig assoziierte Risikofaktoren wie arterielle Hypertonie, Adipositas, Dyslipidämie, Albuminurie und Bewegungsmangel eine große Rolle für das kardiovaskuläre Risiko, wobei die glykämische Kontrolle einen zentralen Faktor darstellt [4, 8, 9]. Insgesamt ist zur Reduktion kardiovaskulärer Ereignisse sowie der Mortalität daher ein multimodaler Therapieansatz entscheidend [1, 10, 11].

Die Interaktionen zwischen Diabetes mellitus und kardiovaskulären Erkrankungen sind vielseitig. So besteht eine hohe Prävalenz von Diabetes mellitus und Glukosestoffwechselstörungen bei kardiovaskulärer Erkrankung mit nachweisbarer Dosis-Wirkungs-Beziehung, wie auch Daten aus Österreich zeigen [12, 13].

Eine moderne, multifaktorielle Therapie bewirkt eine wesentliche Reduktion kardiovaskulärer Komplikationen und der Mortalität, was auch in großen epidemiologischen Analysen dokumentiert wurde [14, 15].

### Diabetes und koronare Herzkrankheit (KHK)

Die koronare Herzkrankheit (KHK) bzw. ischämische Kardiomyopathie ist die führende Ursache für Mortalität bei Menschen mit Diabetes mellitus. Diabetes mellitus ist nicht nur ein wesentlicher Risikofaktor für die Entstehung einer KHK, sondern ist auch bei bereits bestehender KHK mit einem höheren kardiovaskulären Risiko assoziiert. Bei Patient:innen mit Diabetes ist häufiger eine Mehrgefäßerkrankung mit multiplen koronaren Läsionen und vulnerablen Plaques zu verzeichnen, wobei die Qualität der Glukosekontrolle wesentlich das Risiko für Myokardinfarkte bestimmt [1–5, 16–18]. Zudem besteht bei Menschen mit Diabetes mellitus die Gefahr stummer myokardialer Ischämien aufgrund beeinträchtigter Nozizeption [19–21]. Andererseits wird die Diagnose eines bislang unerkannten Diabetes mellitus sowie Prädiabetes häufig im Rahmen eines koronaren Ereignisses gestellt [13, 22]. Zur Reduktion von koronaren Ereignissen und Todesfällen sind ein multifaktorielles Risikomanagement sowie eine moderne Diabetestherapie entscheidend [1, 4, 5, 15].

### Diabetes und periphere arterielle Verschlusskrankheit (pAVK)

Menschen mit Diabetes erkranken im Vergleich zu Menschen ohne Diabetes häufiger und früher an einer peripheren arteriellen Verschlusskrankheit (pAVK). Es werden oft eine raschere Progression und höhere Amputationsraten sowie häufig eine spezifische Morphologie der Gefäßveränderungen beobachtet. Vor allem ein lang bestehender Diabetes und schlechte Glukosekontrolle korrelieren stark mit dem Risiko für pAVK [5, 23].

Begleiterscheinungen des Diabetes mellitus wie die Mediasklerose und eine Mikroangiopathie sowie die periphere diabetische Neuropathie können die Diagnose der pAVK verzögern und den Krankheitsverlauf beschleunigen: Die Neuropathie vermindert das Schmerzempfinden, betroffene Patient:innen werden häufig erst mit schlecht heilenden Wunden oder Druckulzera vorstellig. Ein regelmäßiges aktives Screening auf pAVK ist bei Menschen mit Diabetes wichtig, um frühzeitig das kardiovaskuläre Risikomanagement zu optimieren und auch Lokalproblemen wie dem diabetischen Fußsyndrom und Ulzera vorzubeugen [5, 24, 25].

Von einer Mediasklerose sind hauptsächlich distale Arterien betroffen, deren interventionelle oder chirurgische Behandlung technisch schwieriger und mit höheren Komplikationsraten behaftet ist. Eine Mikroangiopathie verschlechtert auch in Abwesenheit einer Makroangiopathie beziehungsweise nach erfolgreicher Revaskularisation die Wundheilung. Ulzera sowie das diabetische Fußsyndrom und eine höhere Infektionsrate bei Diabetes führen zu einer erhöhten Amputationsrate [5, 23, 25–29]. Die Kombination aus pAVK und Diabetes geht auch mit einem besonders hohen Risiko für kardiovaskulären Tod einher [17, 23].

### Diabetes und zerebrale arterielle Verschlusskrankheit (cAVK)

Die zerebrale arterielle Verschlusskrankheit (cAVK) bzw. Stenosen der A. carotis interna sind bedeutende Auslöser ischämischer Schlaganfälle. Atherosklerotische Veränderungen und Stenosen der hirnzuführenden Gefäße sind bei Diabetes mellitus besonders häufig und mit schlechterer Glukosekontrolle assoziiert. Zudem ist ein bestehender Diabetes mit schwerwiegenderen und komplexeren Veränderungen sowie auch mit höheren Komplikations- und Restenoseraten nach Karotisendarteriektomie sowie nach Stentrevaskularisation der A. carotis assoziiert [24, 30–33].

### Diabetes und abdominelles Aortenaneurysma (AAA)

Diabetes mellitus stellt keinen spezifischen Risikofaktor für die Entwicklung von Aortenaneurysmen dar. Im Gegenteil scheint das Risiko, an einem Aortenaneurysma zu erkranken, bei Menschen mit Diabetes vermindert und die Wachstumstendenz reduziert zu sein [34, 35].

### Diabetes und Herzinsuffizienz

Neben atherosklerotischen kardiovaskulären Erkrankungen ist auch das Risiko von Herzinsuffizienz bei Diabetes mellitus Typ 1 (T1D) und T2D sowie auch bei Prädiabetes deutlich erhöht [5, 13, 36–38]. Das Vorliegen einer Herzinsuffizienz ist mit einer deutlichen Verschlechterung der Prognose verbunden [39]. Die Bedeutung von Diabetes mellitus in der Entstehung von Herzinsuffizienz zeigt sich auch dadurch, dass in Patientenkollektiven von Herzinsuffizienzstudien ein sehr hoher Anteil an Patient:innen mit Diabetes mellitus (oft bis 50 %) eingeschlossen ist [40–43].

Für die Entstehung einer Herzinsuffizienz bei Diabetes mellitus werden traditionell vor allem arterielle Hypertonie sowie KHK bzw. ischämische Kardiomyopathie als Ursache angesehen. Jedoch besteht auch ohne KHK, arterielle Hypertonie oder valvuläre Problematik das Risiko, als Folge von Diabetes mellitus und Prädiabetes eine Herzinsuffizienz zu entwickeln. Faktoren dafür sind unter anderem chronische Inflammationsvorgänge, Insulinresistenz, kapilläre Dysfunktion, myokardiale Fibrosierung und Hypertrophie, mitochondriale Dysfunktion und oxidativer Stress sowie eine kardiovaskuläre autonome Dysfunktion, Lipotoxizität und besonders auch die Glukosetoxizität infolge von Hyperglykämie [36, 44].

Die Bedeutung der Glukosekontrolle in diesem Zusammenhang wird dadurch unterstrichen, dass ein Anstieg des HbA_1c_ um 1 % bei Menschen mit Diabetes mellitus mit einer um 8 % erhöhten Inzidenz für Herzinsuffizienz assoziiert ist [45]. Selbst bei Menschen ohne Diabetes sind höhere HbA_1c_ Werte mit einer erhöhten Inzidenz für Herzinsuffizienz vergesellschaftet [46]. Eine Verdoppelung des HOMA2-IR als Maß für Insulinresistenz ist ebenfalls mit einem um 14 % erhöhten Risiko für Herzinsuffizienz assoziiert [47].

Neben der Genese kann eine Einteilung der Herzinsuffizienz anhand der echokardiographisch bestimmten linksventrikulären Ejektionsfraktion (LVEF) in 3 Kategorien erfolgen [48]. Dies ist praktisch bedeutsam, da sich die Behandlung zwischen den Kategorien unterscheidet.

- Herzinsuffizienz mit erhaltener LVEF (LVEF ≥ 50 %)
  = HFpEF (heart failure with preserved ejection fraction),
- Herzinsuffizienz mit leichtgradig reduzierter LVEF (LVEF 41–49 %)
  = HFmrEF (heart failure with mildly reduced ejection fraction),
- Herzinsuffizienz mit reduzierter LVEF (LVEF ≤ 40 %)
  = HFrEF (heart failure with reduced ejection fraction).

Die Rolle von Diabetes mellitus ist bei der Herzinsuffizienz mit erhaltener Linksventrikelfunktion (HFpEF) besonders groß. Hier beträgt die Prävalenz von Diabetes ca. 45 % [49]. Die Diagnose einer HFpEF wird klinisch sowie durch die Echokardiographie (relevante strukturelle oder funktionelle Abnormalitäten mit diastolischer Dysfunktion bei erhaltener Linksventrikelfunktion [LVEF ≥ 50 %]) und durch Bestimmung von N‑terminalem-proB-Typ natriuretisches Peptid (NT-proBNP > 125 pg/ml) oder BNP (> 35 pg/ml) gestellt [5, 48, 50]. Patient:innen mit vormals reduzierter LVEF ≤ 40 %, welche im Verlauf unter Therapie eine verbesserte LVEF ≥ 50 % aufweisen (HFrecHF) sollen weiterhin wie Patient:innen mit HFrEF behandelt werden [51].

## Kardiovaskuläre Risikostratifizierung und Screening

### Kardiovaskuläre Risikostratifizierung bei Diabetes mellitus

Die Einteilung des kardiovaskulären Risikos bei Menschen mit T2D erfolgt anhand der Leitlinien der ESC Leitlinien zu Diabetes [5] auf Basis einer bestehenden kardiovaskulären Erkrankung, dem Vorhandensein schwerer Endorganschäden sowie des SCORE2-Diabetes-Risikomodells (Tab. 1; [52]). Bezüglich des kardiovaskulären Risikos bei Menschen mit T1D gibt es weitaus weniger Daten, sodass eine formale Kategorisierung des kardiovaskulären Risikos schwieriger ist.

**Tab. 1:** Kardiovaskuläre Risikostratifizierung bei T2D anhand der ESC-Leitlinien zu Diabetes und Prädiabetes 2023. (Mod. nach [5])

Kardiovaskuläre Risikostratifizierung bei Diabetes mellitus Typ 2	
Sehr hohes Risiko	Nachgewiesene kardiovaskuläre Erkrankung
	Schwere Endorganschäden (Nephropathie^*^ oder Kombination aus 3 mikrovaskulären Erkrankungen (z. B. Albuminurie A2 plus Retinopathie plus Neuropathie)
	10-Jahres-Risiko für kardiovaskuläre Erkrankung ≥ 20 % anhand SCORE2-Diabetes
Hohes Risiko	10-Jahres-Risiko für kardiovaskuläre Erkrankung 10–20 % anhand SCORE2-Diabetes
Mittleres Risiko	10-Jahres-Risiko für kardiovaskuläre Erkrankung 5–10 % anhand SCORE2-Diabetes
Niedriges Risiko	10-Jahres-Risiko für kardiovaskuläre Erkrankung < 5 % anhand SCORE2-Diabetes

*SCORE2-Diabetes* Systematic Coronary Risk Estimation 2-diabetes

^*^Definiert als eGFR < 45 ml/min/1,73 m^2^ oder eGFR 45–59 ml/min/1,73 m^2^ und Albuminurie A2 (UACR 30–300 mg/g) oder Albuminurie A3 (UACR > 300 mg/g unabhängig von der eGFR)

Im Hinblick auf Screening und Diagnostik kardiovaskulärer Erkrankungen muss wesentlich zwischen symptomatischen und asymptomatischen Patient:innen unterschieden werden.

Eine individuelle Evaluierung des aktuell vorliegenden kardiovaskulären Risikos soll regelmäßig, mindestens jedoch einmal jährlich durchgeführt werden. Dies beinhaltet neben Anamnese und klinischer Untersuchung nach Möglichkeit und insbesondere bei Vorliegen von Symptomen auch die Messung von NT-proBNP oder BNP zum Screening auf Herzinsuffizienz sowie auch zur Risikostratifizierung für kardiovaskuläre Ereignisse, da gerade niedrige NT-proBNP-Werte < 125 pg/ml mit sehr niedrigem kardiovaskulärem Risiko einhergehen [53]. Eine Einschätzung des kardiovaskulären Risikos kann bei Menschen mit T2D ≥ 40 Jahre auch mit dem Systematic Coronary Risk Estimation 2‑diabetes(SCORE2-Diabetes)-Algorithmus durchgeführt werden [54].

Auch die Bestimmung der Albuminurie mittels Albumin/Kreatinin-Ratio aus dem Spontanharn (UACR) ist notwendig zur Einordnung des kardiovaskulären Risikos [55] und soll zumindest jährlich erfolgen (bei T1D nach zumindest 5 Jahren Krankheitsdauer, bei T2D ab Diagnose), da sich hieraus auch direkte therapeutische Konsequenzen ergeben (Initiierung eines SGLT-2-Hemmers, ACE-Hemmer oder Angiotensin-Rezeptorblocker mit Hochtitration zur maximal verträglichen Dosis, Finerenon sowie nach Möglichkeit Initiierung eines Inkretin-Mimetikums [Semaglutid]) [56, 57].

### KHK-Screening

Bei kardial asymptomatischen Patient:innen ohne für koronare Herzkrankheit (KHK) typische EKG-Veränderungen soll keine weiterführende apparative koronare Abklärung durchgeführt werden [1, 5, 54, 58]. Es kann zwar durch ein CT-basiertes Koronararterien-Kalzium-Scoring eine Risikostratifikation bezüglich kardiovaskulärer Ereignisse durchgeführt werden [59, 60], und auch Screening mittels Myokardszintigraphie detektiert einen relevanten Anteil an Patient:innen mit koronarer Herzkrankheit und stiller Myokardischämie [61]. Allerdings ergibt sich bei optimierter medikamentöser Therapie durch apparatives Screening keine Reduktion an kardialen Ereignissen oder der Gesamtsterblichkeit [62, 63]. Zudem bestand in mehreren Studien keine Überlegenheit eines interventionellen Vorgehens bezüglich Gesamtmortalität bei asymptomatischer KHK gegenüber einer optimierten multifaktoriellen Therapie, wenngleich kardiovaskuläre Ereignisse, kardiovaskulärer Tod sowie bei bestehender Symptomatik auch pektanginöse Beschwerden durch eine Revaskularisationstherapie reduziert werden können [64–69].

Bei symptomatischen Patient:innen im Sinne einer Belastungsdyspnoe, Leistungsverminderung oder bei pektanginösen Beschwerden soll nach Ausschluss eines akuten Koronarsyndroms (ACS) mithilfe von EKG und Troponinbestimmung [70] eine weiterführende Diagnostik je nach Einschätzung der Vortestwahrscheinlichkeit erfolgen. Bei niedriger bis moderater Wahrscheinlichkeit einer obstruktiven KHK hat die koronare CT-Angiographie einen hohen Stellenwert zum Ausschluss („rule out“), während bei höherer Wahrscheinlichkeit funktionelle Tests (Stress-Echo, SPECT, PET oder kardiales MRT) einen hohen Stellenwert zum „rule in“ haben. Bei sehr hoher Wahrscheinlichkeit für eine obstruktive KHK ist direkt eine invasive Koronarangiographie indiziert [54].

### pAVK-Screening

Ein Screening hinsichtlich pAVK soll regelmäßig mittels Anamnese und klinischer Untersuchung (typische Symptome, Pulspalpation, Hautfarbe und -temperatur) sowie bei asymptomatischen Patient:innen mittels ABI-Messung („Ankle-Brachial-Index“ bzw. Knöchel-Arm-Index) erfolgen. Hierbei ist zu beachten, dass Sensitivität und Spezifität dieser Untersuchung mit jeweils um die 80 % besonders bei Patient:innen mit chronischen Wunden als nicht ausreichend anzusehen sind, um eine ursächliche pAVK auszuschließen. Während ein ABI ≤ 0,9 ein guter Hinweis auf eine pAVK ist, können „Normwerte“ > 0,9 bis ≤ 1,4 ebenso die Folge einer Mediasklerose sein wie Werte > 1,4. Letzteres ist besonders bei Diabetes mellitus, aber auch bei chronischer Niereninsuffizienz zu beobachten. Bei betroffenen Patient:innen ist ein < 0,7 reduzierter Toe-Brachial-Index (TBI) häufig ein besserer diagnostischer Hinweis auf eine pAVK. Im Zweifelsfall sollte die Indikation für eine Duplexsonographie der peripheren Arterien großzügig gestellt werden [5, 24, 25, 71, 72]. Bei Patient:innen, die für eine Revaskularisationstherapie infrage kommen, ist eine weiterführende anatomische Bildgebung (Duplexultraschall, CT- oder MR-Angiographie oder intraarterielle digitale Subtraktionsangiographie) empfohlen. Für die Planung einer etwaigen Revaskularisation sollte eine Zuweisung zu einem spezialisierten multidisziplinären Zentrum erfolgen [24, 25, 71–73].

Besonders wichtig zur Prophylaxe des diabetischen Fußsyndroms bei Identifikation eines Risikofußes ist die regelmäßige Inspektion, Schulung der betroffenen Patient:innen sowie deren Angehörigen, das Tragen von geeignetem Schuhwerk sowie die Behandlung von präulzerösen Läsionen (z. B. Hornhautschwielen). Diesbezüglich sei auf das Leitlinienkapitel „Diabetische Neuropathie und diabetischer Fuß (Update 2026)“ verwiesen.

### Screening auf Carotisstenose

In Anlehnung an mehrere internationale Leitlinien ist ein routinemäßiges Screening auf eine eventuell vorliegende asymptomatische Carotisstenose in der Gesamtbevölkerung wie auch bei Patient:innen mit Diabetes ohne weitere Risikofaktoren nicht empfohlen. Jedoch soll bei Personen mit mehr als einem kardiovaskulären Risikofaktor (u. a. andere kardiovaskuläre Erkrankungen, Diabetes mellitus, arterielle Hypertonie, Schlaganfall bei erstgradig Verwandten, Rauchen, vorangehende Bestrahlung im Halsbereich sowie pathologische Strömungsgeräusche über der A. carotis) und entsprechend hoher Vortestwahrscheinlichkeit eine Carotissonographie zum Ausschluss einer signifikanten Stenose zumindest erwogen werden [5, 24, 74, 75].

### Screening auf abdominelles Aortenaneurysma (AAA)

Ein Screening auf ein etwaig vorliegendes AAA mittels Duplexsonographie ist unabhängig vom Vorliegen eines Diabetes bei Männern ≥ 65 Jahren und positiver Raucheranamnese sowie geschlechtsunabhängig ab dem 50. Lebensjahr bei erstgradig Verwandten von Patient:innen mit AAA empfohlen. Ein Screening kann bei Männern ≥ 75 Jahren ohne Nikotinanamnese sowie bei Frauen ≥ 75 Jahren mit aktivem Nikotinkonsum und/oder arterieller Hypertonie erwogen werden [24].

### Screening auf Herzinsuffizienz

Entsprechend den aktuell gültigen Leitlinien der ESC sollte bei Menschen mit Diabetes regelmäßig eine systematische klinische Untersuchung und Anamnese hinsichtlich für Herzinsuffizienz typische Symptome oder Beschwerden (z. B. Atemnot, Orthopnoe, Beinödeme, Gewichtszunahme, reduzierte Belastbarkeit, Fatigue) durchgeführt werden. Bei Vorliegen von Symptomen oder Beschwerden sollen ein 12-Kanal-EKG sowie eine BNP oder NT-proBNP-Bestimmung durchgeführt werden. Erhärtet sich der Verdacht auf eine Herzinsuffizienz, so sollen ein Thoraxröntgen und eine transthorakale Echokardiographie durchgeführt werden [5, 48, 50].

## Kardiovaskuläres Risikomanagement bei T2D und manifester kardiovaskulärer Erkrankung

Eine bereits bekannte kardiovaskuläre Erkrankung wie auch das Vorliegen von Risikofaktoren stellen wesentliche Kriterien für die Auswahl der individuellen Therapie dar. Insbesondere SGLT-2-Hemmer sowie GLP-1-Rezeptoragonisten (GLP‑1 RA) haben hier in mehreren Studien Reduktionen kardiovaskulärer Ereignisse bewirkt.

Neben der medikamentösen antidiabetischen Therapie muss die Wichtigkeit des Lebensstils (Ernährung, Bewegung, Rauchstopp, Alkohol- und Stressreduktion) sowie des Lipid‑, Gewichts- und Blutdruckmanagements betont werden. Diesbezüglich sei auf die entsprechenden Kapitel der Leitlinie verwiesen.

### Metformin

Metformin stellt seit Langem einen unumstrittenen Teil der medikamentösen Erstlinientherapie des T2D dar [76, 77]. Im Langzeit-Follow-up der UKPDS-Studie wurde eine Senkung von Myokardinfarkt und Gesamtmortalität durch Metformin bei übergewichtigen Patient:innen mit T2D beobachtet [78–81]. Auch Metaanalysen konnten einen kardiovaskulären Nutzen von Metformin feststellen, wobei hier Unterschiede abhängig vom jeweiligen Patientenkollektiv und den verwendeten Kontrollsubstanzen bestehen [80, 82–84]. Wichtig ist festzuhalten, dass in sämtlichen moderneren Studien, in denen ein kardiovaskulärer Vorteil neuerer Substanzen (GLP‑1 RA und SGLT-2-Hemmer) gezeigt werden konnte, ein Großteil der Patient:innen Metformin als Basistherapie einnahm [85–87].

### Inkretin-Mimetika

Für die GLP‑1 RA Liraglutid, Semaglutid, Dulaglutid und Tirzepatid ist bei Patient:innen mit Diabetes mellitus Typ 2 eine Senkung kardiovaskulärer Endpunkte belegt [88–90].

In der LEADER-Studie bewirkte Liraglutid eine signifikante Senkung des kardiovaskulären Endpunktes mit vor allem einer Reduktion kardiovaskulärer Todesfälle [88]. Für Semaglutid zeigte die SUSTAIN-6-Studie ebenfalls eine Reduktion kardiovaskulärer Ereignisse, wobei im Besonderen auch nicht tödliche Schlaganfälle seltener auftraten [89]. In der STRIDE-Studie konnten mit Semaglutid zudem eine Verbesserung der Gehstrecke sowie eine Reduktion von Rescue-Therapie und Verbesserung der Lebensqualität bei Patient:innen mit symptomatischer pAVK erreicht werden [91]. Auch für orales Semaglutid konnte eine Reduktion kardiovaskulärer Endpunkte gezeigt werden [92].

Für Dulaglutid wurde in der REWIND-Studie eine Reduktion des kombinierten kardiovaskulären Endpunkts gezeigt [90]. Für Lixisenatid und Exenatid konnte die kardiovaskuläre Sicherheit, jedoch kein substanzspezifischer kardiovaskulärer Zusatznutzen gezeigt werden [93, 94]. In der SURPASS-CVOT-Studie zeigte sich eine kardiovaskuläre Protektion durch Tirzepatid (Nichtunterlegenheit gegenüber Dulaglutid als primärer Endpunkt).

### SGLT-2-Hemmer

Für die SGLT-2-Hemmer Empagliflozin, Dapagliflozin und Canagliflozin konnte bei Patient:innen mit Diabetes mellitus Typ 2 eine Senkung kardiovaskulärer Endpunkte gezeigt werden [87, 95, 96].

Empagliflozin bewirkte in der EMPA-REG OUTCOME-Studie im Vergleich zu Placebo zusätzlich zum blutzuckersenkenden Effekt eine signifikante Reduktion des primären Endpunkts (kardiovaskulärer Tod, nicht tödlicher Myokardinfarkt, nicht tödlicher Insult) bei bereits kardiovaskulär erkrankten Patient:innen. Weiters waren die Gesamtsterblichkeit, die kardiovaskuläre Sterblichkeit sowie Hospitalisierungen aufgrund von Herzinsuffizienz reduziert. Dieser Effekt zeigte sich bereits innerhalb weniger Monate nach Therapiebeginn [97]. Für Canagliflozin wurde in der CANVAS-Studie ebenfalls eine signifikante Senkung des primären Endpunktes (kardiovaskulärer Tod, nicht tödlicher Myokardinfarkt, nicht tödlicher Insult) gezeigt. Dem positiven Resultat steht eine signifikant höhere Amputationsrate vor allem im Fußbereich unter Canagliflozin gegenüber [96]. Dapagliflozin wurde in der DECLARE–TIMI 58-Studie untersucht, wobei hier ca. 59 % der Patient:innen am Beginn der Studie keine kardiovaskuläre Erkrankung aufwiesen. Unter Dapagliflozin bestand eine signifikante Reduktion des kombinierten Endpunktes aus kardiovaskulären Todesfällen und Hospitalisationen aufgrund von Herzinsuffizienz. Dieser Effekt war hauptsächlich durch eine Reduktion der Hospitalisierung durch Herzinsuffizienz getrieben. Die Reduktion von Hospitalisierung wegen Herzinsuffizienz konnte auch in der Gruppe mit multiplen kardiovaskulären Risikofaktoren (Dyslipidämie, Hypertonie, Rauchen) ohne vorhergehende kardiovaskuläre Erkrankung nachgewiesen werden [87].

### Vergleich und Kombination von GLP-1 RA und SGLT-2-Hemmern

Insgesamt lässt sich sagen, dass durch die Verwendung von GLP‑1 RA und SGLT-2-Hemmer bei Patient:innen mit T2D und vorliegender kardiovaskulärer Erkrankung ein vergleichbarer kardiovaskulärer Nutzen erzielt werden kann und durch eine Kombination beider Substanzgruppen additive positive Effekte bestehen, sodass diese beiden Substanzgruppen hier empfohlen sind und auch kombiniert werden sollen [98–100]. Besonders hervorzuheben sind die Reduktion von Hospitalisationen wegen Herzinsuffizienz durch SGLT-2-Hemmer sowie eine Reduktion des Schlaganfallrisikos durch GLP‑1 RA und eine Verbesserung renaler Outcome-Parameter durch beide Substanzklassen [101, 102].

### Pioglitazon

Für Pioglitazon konnte bei Patient:innen mit Diabetes mellitus Typ 2 weder im Vergleich zu Sulfonylharnstoffen (TOSCA-IT-Studie) [103] noch bei Patient:innen mit vorbestehender kardiovaskulärer Erkrankung (PROactive-Studie) eine signifikante Reduktion des primären kardiovaskulären Endpunktes gezeigt werden [104]. Hervorzuheben ist jedoch der positive Effekt von Pioglitazon bei Patient:innen mit vorangegangenem Schlaganfall. In diesem Subkollektiv der PROactive-Studie war das Risiko für einen erneuten Schlaganfall unter Pioglitazon deutlich geringer [105]. Auch bei Patient:innen mit erhöhter Insulinresistenz ohne Diabetes, aber rezent vorangegangenem Insult konnte eine Reduktion eines Rezidivschlaganfalls oder Myokardinfarkts in einer prospektiv randomisierten Studie gezeigt werden [106]. Zu beachten ist, dass Pioglitazon bei bestehender Herzinsuffizienz NYHA I–IV aufgrund des Risikos für gesteigerte Flüssigkeitseinlagerung kontraindiziert ist [107, 108].

### Andere Substanzklassen

Bislang liegen keine Studien vor, die eine eindeutige substanzspezifische Reduktion kardiovaskulärer Endpunkte durch DPP-4-Hemmer oder Sulfonylharnstoffe belegen. Beide Substanzklassen können jedoch als kardiovaskulär sicher eingestuft werden [103, 109].

### Glukosesenkende Therapie im Allgemeinen

Die eindeutige Evidenz des Nutzens einer glukosesenkenden Therapie war lange Zeit auf mikrovaskuläre Komplikationen beschränkt [77, 110]. Verschiedene Interventionsstudien (ACCORD, ADVANCE, VADT) konnten keinen eindeutigen Nutzen einer intensivierten Glukosekontrolle durch die damals verfügbaren antidiabetischen Substanzen (Sulfonylharnstoffe, Insulin, Metformin sowie Glitazone) auf kardiovaskuläre Endpunkte belegen [111–113]. Es konnte jedoch bei Verlängerung des Nachbeobachtungszeitraumes sowie in Metaanalysen eine signifikante Reduktion kardiovaskulärer Ereignisse durch eine intensivierte Glukosekontrolle dokumentiert werden [114, 115].

Ähnliche positive Effekte zeigten sich auch in der UKPDS (T2D) [79] sowie für T1D in der DCCT/EDIC-Studie (T1D) [116]. Zudem konnte in den Studien ein Effekt beobachtet werden, der als „glykämisches Gedächtnis“ („glycemic memory“, „metabolic memory“, „legacy effect“) bezeichnet wird. Darunter versteht man, dass sich Phasen von guter glykämischer Kontrolle langfristig positiv auf das Risiko für kardiovaskuläre Ereignisse auswirken [79, 116–118].

Weiterhin sollte bei der Festlegung der individuellen Therapiestrategie besonderes Augenmerk auf die Vermeidung von Hypoglykämien gelegt werden, da gerade schwere Hypoglykämien mit einem höheren kardiovaskulären Risiko assoziiert sind [119–121].

### Zusammenfassung – Antidiabetische Therapie bei manifester kardiovaskulärer Erkrankung

Zusammenfassend konnte bei Menschen mit T2D durch eine verbesserte Glukoseeinstellung sowie durch zusätzliche substanzspezifische Effekte von Inkretin-Mimetika und SGLT-2-Hemmern eine Senkung kardiovaskulärer Endpunkte gezeigt werden. Zudem bestehen Hinweise auf eine Reduktion kardiovaskulärer Endpunkte durch Metformin sowie für Pioglitazon bei Patient:innen mit rezent vorangegangenem Schlaganfall.

Bei Patient:innen mit bereits bekannter kardiovaskulärer Erkrankung soll eine Substanz mit dokumentierten positiven kardiovaskulären Effekten (SGLT-2-Hemmer und/oder GLP‑1 RA) gemeinsam mit Metformin eingesetzt und eine gute Glukosekontrolle angestrebt werden.

## Kardiovaskuläres Risikomanagement durch antidiabetische Therapie bei T2D und chronischer Nierenerkrankung (CKD)

Patient:innen mit T2D und chronischer Nierenerkrankung (CKD) stellen ein besonderes Kollektiv mit erhöhtem kardiovaskulärem Risiko dar. Sowohl das Ausmaß einer Albuminurie als auch eine erniedrigte GFR korrelieren mit dem kardiovaskulären Risiko [5, 55, 57, 122–124].

### SGLT-2-Hemmer

Bei Patient:innen mit CKD mit einer eGFR > 20 ml/min/1,73 m^2^ soll ein SGLT-2-Hemmer initiiert werden, da hierdurch nierenspezifische wie auch kardiovaskuläre Ereignisse reduziert werden können. Dieser kardiovaskuläre Vorteil besteht unabhängig vom glukosesenkenden Effekt und auch bei Patient:innen mit CKD ohne Diabetes, sodass auch ohne T2D bei CKD ein SGLT-2-Hemmer indiziert ist. Ein bereits etablierter SGLT-2-Hemmer soll bei Verträglichkeit auch bei Absinken der eGFR < 20 ml/min/1,73 m^2^ bis zum Eintreten einer Dialysepflichtigkeit weiter verabreicht werden [55, 125–128]. Für nähere Details sei auf die Leitlinie „Diabetische Nierenerkrankung“ verwiesen.

### Semaglutid

Im FLOW-trial wurde durch Semaglutid bei Patient:innen mit T2D und CKD (eGFR 50–75 ml/min/1,73 m^2^ und UACR 300–5000 mg/g oder eGFR 25–50 ml/min/1,73 m^2^ und UACR 100–5000 mg/g) zusätzlich zu einer Reduktion nierenspezifischer Endpunkte insbesondere auch eine Reduktion kardiovaskulärer Todesfälle sowie der Gesamtmortalität gezeigt [56].

### RAAS-Inhibition und Finerenon

Abseits der glukosesenkenden Therapie ist bei Patient:innen mit T2D und CKD mit Albuminurie eine Therapie mit einem Hemmer des Renin-Angiotensin-Aldosteron-Systems (RAAS; ACE-Hemmer oder Angiotensin-Rezeptorblocker) sowie mit dem nichtsteroidalen Mineralokortikoid-Rezeptor-Antagonisten (nsMRA) Finerenon indiziert [5, 55, 57].

Bei Patient:innen mit T2D und CKD mit Albuminurie (UACR > 30 mg/g, CKD-Stadien G1–G4, A2 und A3) soll eine RAAS-Hemmung mit einem ACE-Hemmer oder Angiotensin-Rezeptorblocker erfolgen. Diese Medikamente sollen bis zur maximal verträglichen Dosis hochtitriert werden [55, 57].

Bei Patient:innen mit T2D und CKD mit persistierender Albuminurie (eGFR > 25 ml/min/1,73 m^2^, normales Serum-Kalium und UACR > 30 mg/g trotz bis zur Verträglichkeitsgrenze hochtritrierten RAAS-Hemmers) ist eine zusätzliche Therapie mit dem nsMRA Finerenon indiziert. Hiermit konnte eine Reduktion nierenspezifischer Endpunkte wie auch eine Reduktion kardiovaskulärer Endpunkte erreicht werden (FIDELIO-DKD, FIGARO-DKD, FIDELITY) [129–132]. Finerenon soll nur bei Serum-Kaliumwerten ≤ 4,8 mmol/l (bzw. ≤ 5 mmol/l mit zusätzlicher Observanz) initiiert werden und soll bei > 5,5 mmol/l pausiert werden [55, 57, 133]. Eine Kombination von Finerenon und SGLT-2-Hemmern bewirkt eine additive UACR-Reduktion (CONFIDENCE-Studie) [134].

Das Vorgehen bei diabetischer Nierenerkrankung über das kardiovaskuläre Risikomanagement hinaus ist im Kapitel „Diabetische Nierenerkrankung“ beschrieben.

## Antidiabetische Therapie bei T2D und Herzinsuffizienz

### SGLT-2-Hemmer

#### Herzinsuffizienz mit erhaltener Linksventrikelfunktion LVEF ≥ 50 % (HFpEF)

Für die SGLT-2-Hemmer Empagliflozin und Dapagliflozin liegen positive Endpunktstudien zur Behandlung der HFpEF vor.

Für Empagliflozin zeigte die EMPEROR-Preserved-Studie bei Patient:innen mit Herzinsuffizienz und LVEF > 40 % eine signifikante Reduktion des primären Endpunktes (kardiovaskulärer Tod oder Hospitalisation aufgrund von Herzinsuffizienz), unabhängig von einem etwaig vorliegenden Diabetes [43]. Für Dapagliflozin zeigte die DELIVER-Studie bei Patient:innen mit Herzinsuffizienz und LVEF > 40 % sowohl bei Patient:innen mit wie auch ohne Diabetes ebenfalls eine signifikante Reduktion des primären Endpunktes (Verschlechterung der Herzinsuffizienz oder kardiovaskulärer Tod) [135].

#### Herzinsuffizienz mit reduzierter Linksventrikelfunktion LVEF ≤ 40 % (HFrEF)

Dapagliflozin, Empagliflozin und Canagliflozin haben in den jeweiligen Endpunktstudien deutliche Reduktionen in der Hospitalisierung aufgrund einer Herzinsuffizienz gezeigt [40, 136, 137].

Für Dapagliflozin wurde in der DAPA-HF-Studie bei Personen mit vorbestehender HFrEF (LVEF < 40 %) und einem NYHA-Stadium ≥ II eine signifikante Reduktion des primären Endpunktes (Verschlechterung der Herzinsuffizienz oder kardiovaskulärer Tod) gezeigt. Dieser Effekt war unabhängig davon, ob ein Diabetes mellitus Typ 2 bestand oder nicht [136]. In der EMPEROR-Reduced-Studie führte die Therapie mit Empagliflozin bei Menschen mit manifester HFrEF (NYHA II–IV und LVEF ≤ 40 %) unabhängig vom Vorliegen eines Diabetes mellitus zu einer signifikanten Reduktion des primären Endpunkts (Hospitalisierung aufgrund von Herzinsuffizienz oder kardiovaskulärer Tod) [40]. In allen Studien war auch ein großer Anteil an Patient:innen mit Prädiabetes inkludiert.

### Weitere antidiabetische Medikamentenklassen

#### Metformin

Für Metformin liegt keine prospektiv randomisierte Studie bei Patient:innen mit T2D und Herzinsuffizienz vor. In retrospektiven Datenanalysen und Observationsstudien konnte bei mit Metformin behandelten Patient:innen mit T2D und Herzinsuffizienz eine geringere Mortalität beobachtet werden [138–140]. Die früher bestehenden Bedenken einer erhöhten Gefahr einer sehr seltenen Laktatazidose unter Metformin haben sich bei chronischer Herzinsuffizienz nicht bewahrheitet, und auch der Zulassungstext von Metformin wurde im Jahr 2006 entsprechend geändert. Bei akuter und hämodynamisch instabiler Herzinsuffizienz muss dieses Risiko jedoch berücksichtigt werden [141, 142].

#### Pioglitazon

Pioglitazon ist laut Zulassungstext bei Herzinsuffizienz NYHA I–IV aufgrund des Risikos einer gesteigerten Flüssigkeitseinlagerung kontraindiziert und darf daher bei manifester Herzinsuffizienz generell nicht eingesetzt werden [107].

#### Dipeptidylpeptidase-IV(DPP-IV)-Hemmer

In den kardiovaskulären Endpunktstudien der Dipeptidylpeptidase-IV(DPP-IV)-Hemmer wurde in der SAVOR-TIMI 53-Studie ein 27 % höheres Risiko für die Hospitalisation aufgrund einer Herzinsuffizienz unter Saxagliptin beobachtet [143]. In EXAMINE (Alogliptin), CARMELINA (Linagliptin) und TECOS (Sitagliptin) konnte diese Assoziation jedoch nicht beobachtet werden [144].

#### Inkretin-Mimetika

Für die am Markt befindlichen GLP‑1 RA konnte bislang nur in speziell definierten Kollektiven ein positiver Effekt hinsichtlich herzinsuffizienzspezifischer Endpunkte bei Patient:innen mit T2D gezeigt werden. Die GLP‑1 RA Liraglutid (LEADER-Studie), Dulaglutid (REWIND) und Semaglutid (SUSTAIN‑6; orales Semaglutid: SOUL) zeigten bei Personen mit T2D und hohem kardiovaskulärem Risiko keine Reduktion von Herzinsuffizienzendpunkten und scheinen, was diese anbelangt, also neutral zu sein [88–90, 92]. Die Sicherheit bezüglich kardiovaskulärer und auch herzinsuffizienzspezifischer Endpunkte von Liraglutid und Dulaglutid und Semaglutid konnte jedoch auch spezifisch für Patient:innen mit T2D und Herzinsuffizienz gezeigt werden [145–147]. Für Patient:innen mit T2D und CKD zeigte sich im FLOW-trial mit Semaglutid eine Reduktion von Herzinsuffizienzendpunkten [147].

Bei Personen mit Adipositas (BMI ≥ 30) und HFpEF (eingeschlossen waren hier Patient:innen mit einer LVEF ≥ 45 %) konnte ein Vorteil durch höher dosiertes Semaglutid 2,4 mg gezeigt werden. So konnten in diesem Kollektiv sowohl bei Personen mit (STEP-HFpEF DM) wie auch ohne T2D (STEP-HFpEF) Herzinsuffizienzsymptome signifikant reduziert werden [148, 149]. In einer gepoolten Post-hoc-Analyse bestand auch eine Reduktion des kombinierten Endpunkts aus kardiovaskulärem Tod und Herzinsuffizienzereignissen [150].

Auch für den kombinierten GIP/GLP‑1 RA Tirzepatid konnten in der SUMMIT-Studie bei Patient:innen mit HFpEF und Adipositas (BMI ≥ 30) eine Reduktion des primären Endpunktes (kardiovaskulärer Tod oder Verschlechterung der Herzinsuffizienz) wie auch eine symptomatische Verbesserung gezeigt werden. Bei knapp der Hälfte der Studienteilnehmer bestand auch ein Diabetes [151].

### Finerenon

In der FINEHEARTS-HF-Studie konnte durch den Einsatz von Finerenon bei Patient:innen mit Herzinsuffizienz mit LVEF ≥ 40 % (HFpEF und HFmrEF) eine Reduktion des kombinierten primären Endpunkts (Verschlechterung einer Herzinsuffizienz sowie kardiovaskulärer Tod) erreicht werden [152]. In dieser Studie waren Menschen mit und ohne Diabetes sowie Prädiabetes eingeschlossen. Während Finerenon in den USA zur Therapie bei Patient:innen mit Herzinsuffizienz und einer LVEF ≥ 40% bereits zugelassen ist, ist die Zulassung in Europa noch ausständig.

### Zusammenfassung – Antidiabetische Therapie bei T2D und Herzinsuffizienz

Bei Vorliegen einer manifesten Herzinsuffizienz soll unabhängig von der LVEF (also bei HFpEF, HFmrEF und HFrEF) und unabhängig vom HbA_1c_ bei Patient:innen mit und ohne Diabetes mellitus eine Therapie mit einem SGLT-2-Hemmer etabliert werden [5, 48, 50].

Bei HFrEF sind unabhängig von einem etwaig vorliegenden Diabetes mellitus zusätzlich ein ACE-Hemmer oder der Angiotensin-Rezeptor-Neprilysin-Inhibitor (ARNI) Sacubitril/Valsartan (bzw. bei Unverträglichkeit alternativ ein Angiotensin-Rezeptorblocker) sowie ein Betablocker und ein steroidaler Mineralokortikoid-Rezeptor-Antagonist (MRA) indiziert [48].

Bei HFpEF und HFmrEF ist unabhängig von einem etwaig vorliegenden Diabetes mellitus der Einsatz von Finerenon empfohlen, sobald das Präparat in dieser Indikation auch in Europa zugelassen ist [152].

Bei HFpEF und Adipositas ist unabhängig von einem etwaig vorliegenden Diabetes mellitus eine Therapie mit dem GLP‑1 RA Semaglutid oder dem GIP/GLP‑1 RA Tirzepatid empfohlen [148, 149, 151].

## Thrombozytenaggregationshemmung bei Diabetes

### Therapieindikationen und Nutzen-Risiko-Abwägung

Eine medikamentöse Hemmung der Thrombozytenaggregation reduziert kardiovaskuläre Morbidität und Mortalität bei Patient:innen mit manifester kardiovaskulärer Erkrankung sowie mit hohem kardiovaskulärem Risiko. Diesem möglichen Nutzen muss jedoch die Rate an Blutungskomplikationen gegenübergestellt werden. Diese sind sehr stark abhängig vom untersuchten Kollektiv [153–155]. Das Blutungsrisiko unter Acetylsalicylsäure (ASS) ist altersabhängig und sowohl für intrakranielle Blutungen [156] als auch für gastrointestinale Blutungen [157] erhöht.

#### Sekundärprävention

Eine klare Indikation zur Thrombozytenaggregationshemmung besteht bei Personen mit klinisch manifester kardiovaskulärer Erkrankung (Sekundärprävention). Diese Patient:innen sollten bei Abwesenheit anderer blutverdünnender Medikation ASS (75–100 mg täglich) oder bei dokumentierter ASS-Allergie Clopidogrel (75 mg täglich) erhalten [5, 54, 158].

#### Primärprävention

Bei Patient:innen ohne etablierte kardiovaskuläre Erkrankung (Primärprävention) besteht nach Abwägung zwischen Risikoreduktion kardiovaskulärer Ereignisse und erhöhtem Blutungsrisiko kein klarer Nutzen einer Thrombozytenaggregationshemmung. Für einzelne Patientengruppen mit sehr hohem kardiovaskulärem Risiko und geringem Blutungsrisiko scheint jedoch ein Nutzen einer Thrombozytenaggregationshemmung möglich. Eine generelle Empfehlung zur Thrombozytenaggregationshemmung zur Primärprävention kardiovaskulärer Ereignisse kann auf Basis der vorliegenden Daten nicht ausgesprochen werden, sodass eine individualisierte Herangehensweise gewählt werden und für Patient:innen mit hohem Risiko eine Thrombozytenaggregationshemmung erwogen werden kann [5, 144, 159–165].

#### Antazide Therapie

Das gastrointestinale Blutungsrisiko sowie die Entwicklung gastroduodenaler Ulzera kann durch eine antazide Therapie reduziert werden [166, 167], wobei diesbezüglich Protonenpumpenhemmer (PPI) anderen Präparaten wie H_2_-Blockern überlegen sind [168]. Die Studienlage bezüglich des Vorteils einer PPI-Therapie bei Einnahme von ASS ist inhomogen und die Datenlage schwach [169], und klare Empfehlungen anderer Gesellschaften für Patient:innen mit ASS-Monotherapie existieren bislang nicht. Für Patient:innen mit hohem Blutungsrisiko und ASS-Therapie scheint jedoch eine PPI-Therapie von Vorteil zu sein [170, 171].

Insgesamt soll für Patient:innen mit hohem Blutungsrisiko (z. B. Begleittherapie mit NSAR, Cortison, Antikoagulanzien, anderen Thrombozytenaggregationshemmern oder SSRI; Alter ≥ 65 Jahre; Zustand nach oberer GI-Blutung; Dyspepsie; gastroösophageale Refluxkrankheit [GERD]; chronischer Alkoholabusus [5]) eine PPI-Therapie begleitend zur Thrombozytenaggregationshemmung zur Reduktion des gastrointestinalen Blutungsrisikos erwogen werden.

### Zusammenfassung – Thrombozytenaggregationshemmung bei Diabetes

Patient:innen mit klinisch manifester kardiovaskulärer Erkrankung sollen bei Abwesenheit anderer blutverdünnender Medikation zur Sekundärprävention ASS (75–100 mg täglich) oder bei dokumentierter ASS-Allergie Clopidogrel (75 mg täglich) erhalten.

Für Patient:innen mit hohem oder sehr hohem kardiovaskulärem Risiko ohne bestehende kardiovaskuläre Erkrankung kann eine Thrombozytenaggregationshemmung unter Berücksichtigung der individuellen Situation sowie von Kontraindikationen zur Primärprävention erwogen werden. Für Patient:innen mit mittlerem und niedrigem Risiko ist eine Thrombozytenaggregationshemmung zur Primärprävention kardiovaskulärer Ereignisse nicht empfohlen.

Für Patient:innen mit hohem Blutungsrisiko soll begleitend zur Thrombozytenaggregationshemmung eine PPI-Therapie erwogen werden.

## Kardiovaskuläre Erkrankungen und Herzinsuffizienz bei Diabetes mellitus Typ 2 – Empfehlungen zu Diagnostik und Management

- Eine **Evaluierung des kardiovaskulären Risikos soll zumindest jährlich **durchgeführt werden inklusive **Anamnese** und **klinischer Untersuchung** sowie nach Möglichkeit Bestimmung von **(NT-pro)BNP, eGFR** und **UACR**. Die kardiovaskuläre Risikostratifizierung bei Diabetes mellitus Typ 2 kann anhand des **SCORE2-Diabetes** sowie etwaiger bestehender kardiovaskulärer Erkrankung bzw. schwerer Endorganschäden erfolgen.
- **KHK Screening:**
  - Bei kardial asymptomatischen Patient:innen ohne KHK-typische EKG-Veränderungen soll keine weiterführende apparative koronare Abklärung durchgeführt werden.
  - Bei symptomatischen Patient:innen (z. B. Belastungsdyspnoe, zunehmender Leistungsverminderung, pektanginöse Beschwerden) soll nach ACS-Ausschluss eine weiterführende Diagnostik je nach Vortestwahrscheinlichkeit durchgeführt werden:
    - bei niedriger Vortestwahrscheinlichkeit: „rule out“ z. B. mit koronarer CT-Angiographie,
    - bei hoher Vortestwahrscheinlichkeit: funktionelle Tests zu bevorzugen (Stress-Echo, SPECT, PET, kardiales MRT),
    - bei sehr hoher Wahrscheinlichkeit für eine obstruktive KHK ist direkt eine invasive Koronarangiographie indiziert.
- **Screening auf pAVK, Carotisstenose und AAA:**
  - Ein Screening auf pAVK soll regelmäßig mittels Anamnese/klinischer Untersuchung sowie bei asymptomatischen Patient:innen mittels ABI-Messung erfolgen. Bei inkonklusiver Konstellation sollen niederschwellig eine TBI-Messung sowie eine Duplexsonographie der peripheren Arterien durchgeführt werden.
  - Ein routinemäßiges Screening auf eine asymptomatische Carotisstenose bei Menschen mit Diabetes ohne weitere kardiovaskuläre Risikofaktoren ist nicht empfohlen. Bei mehr als einem kardiovaskulären Risikofaktor soll eine Carotissonographie erwogen werden.
  - Ein Screening auf ein AAA mittels Duplexsonographie ist bei Männern ≥ 65 Jahren und positiver Raucheranamnese sowie bei erstgradig Verwandten ≥ 50 Jahren von Patient:innen mit AAA empfohlen.
- **Bekannte kardiovaskuläre Erkrankung:**
  - Bei Patient:innen mit bereits bekannter kardiovaskulärer Erkrankung sollen **SGLT-2-Hemmer** und/oder **Inkretin-Mimetika** gemeinsam mit **Metformin** eingesetzt werden.
  - Bei Patient:innen **nach kardiovaskulärem Ereignis** soll eine Thrombozytenaggregationshemmung mit **Acetylsalicylsäure** (ASS, 75–100 mg/d) zur Sekundärprävention etabliert werden (alternativ kann bei Unverträglichkeit Clopidogrel 75 mg/d verwendet werden).
  - Für Menschen mit T2D und hohem oder sehr hohem Risiko ohne bestehende kardiovaskuläre Erkrankung kann eine Thrombozytenaggregationshemmung unter Berücksichtigung der individuellen Situation, des Blutungsrisikos sowie von Kontraindikationen zur Primärprävention erwogen werden.
- **Herzinsuffizienz:**
  - Ein **Screening auf Herzinsuffizienz** (klinische Untersuchung und Anamnese sowie nach Möglichkeit Bestimmung von (NT-pro)BNP) soll regelmäßig durchgeführt werden. Bei Symptomen (z. B. Atemnot, Beinödeme, Fatigue) sollen ein 12-Kanal-EKG sowie eine (NT-pro)BNP-Bestimmung gefolgt von einem Thoraxröntgen und bei Auffälligkeiten einer transthorakalen Echokardiographie durchgeführt werden.
  - Bei Vorliegen einer **manifesten Herzinsuffizienz** soll unabhängig von der LVEF (also bei HFpEF, HFmrEF und HFrEF) und unabhängig vom HbA_1c_ eine Therapie mit einem **SGLT-2-Hemmer** etabliert werden.
  - Bei **HFrEF** sind zusätzlich ein **ACE-Hemmer oder ARNI** (bzw. bei Unverträglichkeit alternativ ein Angiotensin-Rezeptorblocker) sowie ein **Betablocker** und ein **MRA** indiziert.
  - Bei **HFpEF und HFmrEF** ist unabhängig von einem etwaig vorliegenden Diabetes mellitus der Einsatz von **Finerenon** empfohlen, sobald das Präparat in dieser Indikation auch in Europa zugelassen ist.
  - Bei HFpEF und Adipositas ist eine Therapie mit dem GLP‑1 RA Semaglutid oder dem GIP/GLP‑1 RA Tirzepatid empfohlen.
- **Chronische Nierenerkrankung (CKD):**
  - **Screening:** Die Bestimmung der **eGFR** sowie der Albuminurie (**UACR** aus dem Spontanharn) soll **zumindest jährlich** erfolgen (UACR-Screening bei T1D nach 5 Jahren Krankheitsdauer, bei T2D ab Diagnose).
  - Bei Patient:innen mit CKD ist eine Therapie mit einem **SGLT-2-Hemmer** sowie bei Albuminurie zusätzlich mit einem RAAS-Hemmer und bei UACR > 30 mg/g trotz RAAS-Hemmer **Finerenon** indiziert.
  - Bei Patient:innen mit CKD soll ein **Inkretin-Mimetikum** gemeinsam mit **Metformin** (bei eGFR > 30 ml/min/1,73 m^2^) eingesetzt werden.

**Abb. 1:**
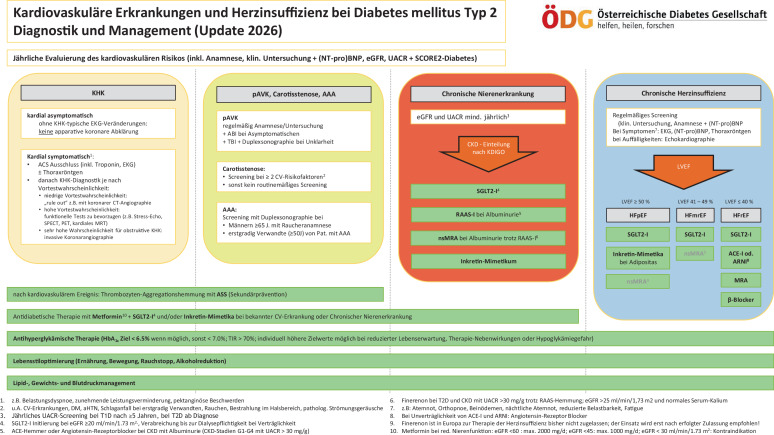
Kardiovaskuläre Erkrankungen und Herzinsuffizienz bei Diabetes mellitus Typ 2 – Diagnostik und Management. *Grau hinterlegte Felder* stellen Diagnosen und Erkrankungen dar. *Weiß hinterlegte Felder* stellen diagnostische Maßnahmen und Screening-Empfehlungen dar. *Grün hinterlegte Felder* stellen therapeutische Maßnahmen und Empfehlungen dar. *AAA* abdominelles Aortenaneurysma, *ABI* Ankle-Brachial-Index, *ACE‑I* Angiotensin-converting-enzyme-Inhibitor, *aHTN* arterielle Hypertonie, *ASS* Acetylsalicylsäure, *cAVK* zerebrale arterielle Verschlusskrankheit, *CKD* chronische Nierenerkrankung, *ARNI* Angiotensin-Rezeptor-Neprilysin-Inhibitor, *CV* kardiovaskulär, *DM* Diabetes mellitus, *eGFR* „estimated glomerular filtration rate“, *EKG* 12-Kanal-EKG, *Echokardiographie* transthorakale Echokardiographie, *HFmrEF* „heart failure with mildly reduced ejection fraction“, *HFpEF* „heart failure with preserved ejection fraction“, *HFrEF* „heart failure with reduced ejection fraction“, *KHK* koronare Herzkrankheit, *MRA* Mineralokortikoid-Rezeptor-Antagonist, *MRT* Magnetresonanztomographie, *nsMRA* nichtsteroidaler Mineralokortikoid-Rezeptor-Antagonist, *(NT-pro)BNP* (N-terminales-pro)B-Typ natriuretisches Peptid, *PET* Positronenemissionstomographie, *SGLT2‑I* Sodium-glucose-co-transporter 2-Inhibitor, *SPECT* Single-Photon-Emissions-Computertomographie, *TBI* Toe-Brachial Index, *TIR* „time in range“, *UACR* Albumin/Kreatinin-Ratio aus dem Spontanharn

Diese Empfehlungen sind auch in Abb. 1 zusammengefasst.

## Interessenkonflikt

M.W. Heinzl gibt folgende Interessenkonflikte an: Honorare sowie Kongressunterstützung von Novo Nordisk, Boehringer Ingelheim, AstraZeneca, Eli Lilly, Novartis, Sanofi-Aventis, Amgen und Bayer erhalten. M. Resl hat von folgenden Unternehmen, die teils auch fördernde Mitglieder der ÖDG sind, Honorare für Vorträge und/oder Beratertätigkeit bzw. Fortbildungsunterstützung erhalten hat: Boehringer Ingelheim, Dexcom, Abbott, Eli Lilly. C. Steinwender gibt folgende Interessenkonflikte an: Vorträge und Advisory Boards für Medtronic, Biotronik, Abbott und Boston Scientific. P. Fellinger gibt folgende Interessenkonflikte an: Kongressunterstützung von Eli Lilly, Novartis und Sanofi-Aventis erhalten. T. Steinmaurer gibt folgende Interessenkonflikte an: Honorare sowie Kongressunterstützung von Novo Nordisk, Boehringer Ingelheim, AstraZeneca, Eli Lilly, Novartis, Sanofi-Aventis, Amgen und Mylan erhalten. C.H. Saely hat von folgenden Firmen Honorare für Vorträge, Beratungstätigkeit und Forschungsunterstützung erhalten: Amgen, AstraZeneca, Bayer, Boehringer Ingelheim, Eli Lilly, Genericon, Genzyme, Merck, MSD, Novartis, Novo Nordisk, Omega Pharma, Pfizer, Sanofi. F. Hoppichler gibt folgende Interessenkonflikte an: Honorare und Projektförderungen von Boehringer Ingelheim, AstraZeneca, Eli Lilly, Sanofi-Aventis und Novo Nordisk erhalten. H. Stingl hat von folgenden Unternehmen, die teils auch fördernde Mitglieder der ÖDG sind, Honorare für Vorträge und/oder Beratertätigkeit bzw. Fortbildungsunterstützung erhalten: Abbott, Amarin, Amgen, AstraZeneca, Boehringer Ingelheim, Daiichi Sankyo, Eli Lilly, Novo Nordisk, Novartis, Sanofi Aventis, Viatris. T.C. Wascher gibt folgende Interessenkonflikte an: Forschungsunterstützungen und/oder Honorare von Amgen, AstraZeneca, Bayer Austria, Eli Lilly, MSD, Novartis, Novo Nordisk, Sanofi, Daiichi-Sankyo. Y. Winhofer hat von folgenden Unternehmen, die teils auch fördernde Mitglieder der ÖDG sind, Honorare für Vorträge und/oder Beratertätigkeit bzw. Fortbildungsunterstützung erhalten: Abbott, Amgen, AstraZeneca, Bayer Healthcare, Boehringer Ingelheim, Daiichi Sankyo, Dexcom, Eli Lilly, Novartis, Novo Nordisk, Sanofi Aventis. H. Sourij hat von folgenden Unternehmen Honorare für Vorträge oder Beratertätigkeit erhalten: Amarin, Amgen, AstraZeneca, Boehringer-Ingelheim, Bayer, Daiichi Sankyo, Eli Lilly, Novo Nordisk, Novartis, Sanofi-Aventis. Forschungsförderungen (an die MedUni Graz): Amarin, Boehringer Ingelheim, Eli Lilly und Daiichi Sankyo. B. Paulweber gibt folgende Interessenkonflikte an: Honorare sowie Kongressunterstützung von Amgen, AstraZeneca, Boehringer Ingelheim, Novartis, Sanofi-Aventis und Sobi erhalten. H. Toplak gibt folgende Interessenkonflikte an: Honorare für Beratung und Vortragstätigkeit von Amgen, Böhringer Ingelheim, Daiichi Sankyo, Mylan, Novartis, Novo Nordisk, Sanofi; Forschungsunterstützung von Boehringer Ingelheim, Novartis und Novo Nordisk. H. Drexel gibt folgende Interessenkonflikte an: Advisory boards sowie consultancy bei Daiichi Sankyo, Novartis und MedMedia; Vorträge und Vorsitze bei Menarini, Sanofi und Novartis. T. Stulnig gibt folgende Interessenkonflikte an: Reisekostenunterstützung und Honoraria von Akcea, Alexion, Amgen, AstraZeneca, Biomarin, Boehringer Ingelheim, Daiichi Sankyo, Eli Lilly, Novartis, Novo Nordisk, Sanofi; Forschungsunterstützung von 89bio, Akcea, Amgen, Boehringer Ingelheim, Daiichi Sankyo, Eli Lilly, Novartis, Sanofi. M. Clodi gibt folgende Interessenkonflikte an: Vorträge, Studien und Beratung für Novo Nordisk, Eli Lilly, Boehringer Ingelheim, AstraZeneca, Novartis, Daiichi Sankyo, Amgen, Wörwag, Sanofi, Amarin, Roche, Bayer. J. Maier, J. Falkensammer und B. Föger geben an, dass kein Interessenkonflikt besteht.
